# Relationship between psychological characteristics, personality traits, and training on performance in a neonatal resuscitation scenario: A machine learning based analysis

**DOI:** 10.3389/fped.2022.1000544

**Published:** 2022-11-18

**Authors:** V. Giordano, K. Bibl, A. Felnhofer, O. Kothgassner, P. Steinbauer, F. Eibensteiner, P. Gröpel, F. Scharnowski, M. Wagner, A. Berger, M. Olischar, D. Steyrl

**Affiliations:** ^1^ Division of Neonatology, Pediatric Intensive Care and Neuropediatrics, Department of Pediatrics, Comprehensive Centre for Paediatrics (CCP), Medical University of Vienna, Vienna, Austria; ^2^ Division of Pediatric Pulmonology, Allergology and Endocrinology Medicine Department of Pediatrics and Adolescent Medicine, Comprehensive Centre for Paediatrics (CCP), Medical University of Vienna, Vienna, Austria; ^3^ Department of Child and Adolescent Psychiatry, Comprehensive Centre for Paediatrics (CCP), Medical University of Vienna, Vienna, Austria; ^4^ Department of Emergency Medicine, Medical University of Vienna, Vienna, Austria; ^5^ Division of Sport Psychology, Centre for Sport Science and University Sports, University of Vienna, Vienna, Austria; ^6^ Department of Cognition, Emotion, and Methods in Psychology, University of Vienna, Vienna, Austria

**Keywords:** resuscitation, simulation, stress, training, psychological traits

## Abstract

**Background:**

In life-threatening emergency events, prompt decision-making and accurate reactions are essential for saving a human's life. Some of these skills can be improved by regular simulation trainings. However, besides these factors, individual characteristics may play a significant role in the patients' outcome after a resuscitation event. This study aimed to differentiate personality characteristics of team members who take responsibility for their actions, contextualizing the effect of training on resuscitation performance.

**Methods:**

Six hundred and two third-year medical students were asked to answer psychological and personality questionnaires. Fifty-five of them performed in a neonatal simulation resuscitation scenario. To assess participants' performances in the NLS scenario, we used a scenario-based designed NLS checklist. A machine learning design was utilized to better understand the interaction of psychological characteristics and training. The first model aimed to understand how to differentiate between people who take responsibility for their actions vs. those who do not. In a second model, the goal was to understand the relevance of training by contextualizing the effect of training to other important psychological and personality characteristics like locus of control, anxiety, emotion regulation, openness to experience, conscientiousness, extraversion, agreeableness, and neuroticism.

**Results:**

No statistically significant differences were found for psychological characteristics between the training group and the no training group. However, as expected, differences were noted in favor of the training group for performance and within gender for psychological characteristics. When correcting for all these information in a model, anxiety and gender were the most important factors associated with taking responsibility for an action, while training was the only relevant factor in explaining performance during a neonatal resuscitation scenario.

**Conclusion:**

Training had a significantly stronger effect on performance in medical students in a neonatal resuscitation scenario than individual characteristics such as demographics, personality, and trait anxiety.

## Background

Healthcare professionals' performance during emergency situations, is determined by many internal and external factors (1). Clearly, the medical expertise and experience, as well as technical skills of healthcare professionals, play an important role. Furthermore, environmental factors such as setting, surroundings (1, 2), and medical equipment (3) also have a significant impact on providers' performance during emergencies and thus, the patients' outcome. However, mastering emergency situations may not only be linked to the above-mentioned factors, but may also depend on other factors such as group dynamics (e.g., teamwork, leadership, team hierarchy) (4), and personal mental state (e.g., fatigue, stress) (5, 6). It is therefore reasonable that performers' personality traits and anxiety also can affect performance during emergencies situations. In this study, we examined the impact of personality traits, anxiety, and training on the performance in a demanding medical care training scenario for critically ill newborns in the delivery room (6). We chose this scenario as there is evidence that health care professionals estimate emergencies as being even more challenging when children are involved (7). Extensive resuscitation after birth is a rare event with fewer than 0.3% of all deliveries (6, 8, 9), and therefore the Newborn Life Support (NLS) algorithm needs to be trained in simulation settings on a regular basis. It has been shown that trainees' stress levels are increased in a simulated resuscitation setting as they are in real settings (10), and that high stress in healthcare providers worsens cognitive processes (e.g., attention, working memory, decision making) and consequently lead to poor patient outcomes (11).

According to LeBlanc (11), the initial stress response in an acute stress situation results from the interaction of the demands of an individual's environment and the person's resources to meet the demands. However, this response is potentially moderated by individual characteristics (12–14). Within this mediating process, the construct of “*Locus of Control*” may play a major role in identifying people who actively take responsibility for their actions vs. those who attribute failures in their life to external causes (13). In order to understand the specific interaction of psychological characteristics and personality traits in relation to the effect of training, we aim at investigating the following research questions by using a robust machine learning based multivariate analysis design:

What are the individual characteristics that describe those people who are more willing to take responsibility for their actions?
What is the effect of psychological characteristics (including the willingness to take responsibility for one's actions), personality traits, and training on performance in a neonatal resuscitation scenario?

Finally, based on previous literature showing differences in personality traits between genders, this information was integrated in our analysis.

## Material and methods

### Procedure

This analysis is part of a brighter project aiming at investigating the link between stress and performance in a simulated neonatal resuscitation scenario including measurements of salivary cortisol and heart rate (“Coping with stress: A multi-method study on the link between stress and performance in a neonatal simulation scenario”). This study was performed at the Pediatric Simulation Center of the Medical University of Vienna (MUV) from December 2018 to August 2020. We recruited third-year medical students during their participation in the obligatory Pediatric Basic Life Support (PBLS) course during their medical education. This mandatory workshop provides students a solid set of knowledge of the PBLS guidelines, but without including the Newborn Life Support (NLS) algorithm. Since there is no course in the curriculum of the MUV providing NLS training, we considered all students as non-experienced in terms of neonatal life support at the time of recruitment. The local ethics committee (1277/2018) approved the study, and every student signed a written informed consent prior to study participation.

In a first step, all students who agreed to participate were asked to answer a brief anamnestic section and different standardized questionnaires (section: Questionnaire screening) to evaluate their psychological characteristics and personality traits. In a second step, students were contacted *via* mail in order to perform in an unknown simulated neonatal resuscitation scenario. Participants in this subgroup were randomly allocated to either the training group or the control group. While in the training group participants received a practical NLS training twice prior to performance assessment, the control group did not take part in any training. Subsequently, each student's performance was assessed in an unknown neonatal resuscitation scenario that lasted up to ten minutes. All scenarios were recorded and stored using audio-visual recording by SimStation (SIMCharacters GmbH, Vienna, Austria). Two independent experts in the field of neonatology and simulation scored each participant's performance using a scenario-based checklist, which includes all relevant steps for a guideline-concordant medical care of a critically ill newborn.

### Questionnaire screening

All students who gave consent to participate were asked to complete a short questionnaire on age, gender, and previous participation to an NLS training. In a next step, participants answered the following questionnaires on personality characteristics:

#### State-trait anxiety inventory (STAI)

The STAI is a psychological questionnaire based on a four-point Likert scale (almost never, sometimes, often, almost always) consisting of two different parts: the state anxiety inventory and the trait anxiety inventory. Each scale consists of twenty items. The trait anxiety inventory measures feelings of stress, worry, and discomfort that one experiences on a day-to-day basis in typical everyday life situations. Scores range from twenty to eighty, with higher scores correlating with more trait anxiety. In this work, we used the STAI trait total score, also further recognized as “*trait anxiety*” through the rest of the manuscript (15).

#### Questionnaire for measurement of competence and control orientation – “Locus of control” (German version – Fragebogen zu Kompetenz- und Kontrollüberzeugungen FKK)

The questionnaire on competence and control beliefs is a tool for adults and adolescents composed of thirty-two items, covering the following psychological constructs: (a) generalized self-concept of one's own abilities; (b) internal control beliefs (the way a person attributes to himself the causes of an action and takes responsibility); (c) social externality (the belief that others control one's fate) and (d) fatalistic externality (both related to the concept of attributing the consequences of an action to fate or other). In this work, we used the internality vs. externality control belief (FKK) score, further referred in text as “*control beliefs*” (13).

#### Emotion regulation questionnaire (ERQ)

The Emotion Regulation Questionnaire (16, 17) is one of the first validated instruments for the investigation of emotion regulation processes. It consists of ten items on a 7-point Likert-type scale ranging from 1 (strongly disagree) to 7 (strongly agree). The questionnaire is an easy tool that takes little time effort and investigates two common regulation strategies: expressive suppression (changing the way one behaviorally responds to emotion-eliciting events) and cognitive reappraisal (changing the way one thinks about potentially emotion-eliciting events) (12). In this work, we used both scores (ERQ cognitive reappraisal, ERQ expressive suppression).

#### Big five-inventory-10 (BFI-10)

The Big Five inventory is a valid and reliable tool to describe common traits of human personality (14). It includes five factors: openness to experience, conscientiousness, extraversion, agreeableness, and neuroticism (18).

• Openness to experience (inventive/curious vs. consistent/cautious): reflects the degree of intellectual curiosity, creativity, and a preference for novelty and variety a person has. High openness can be perceived as unpredictability and related to a way of thinking outside conventional schemes. Conversely, people with low openness seek to gain fulfillment through perseverance and are characterized as pragmatic, data-driven, or closed-minded (19, 20).
• Conscientiousness (efficient/organized vs. easy-going/careless): describes differences in self-discipline between people who prefer planned rather than spontaneous behavior.
• Extraversion: (outgoing/energetic vs. solitary/reserved): is described by items like energy, positive emotions, and sociability. High extraversion is often perceived as attention-seeking and domineering. Low extraversion causes a reserved, reflective personality, which can be perceived as aloof.
• Agreeableness (friendly/compassionate vs. challenging/detached): reflects the tendency to be compassionate and cooperative rather than suspicious and antagonistic towards other.
• Neuroticism (sensitive/nervous vs. secure/confident): is an index of emotional stability and impulse control that reflects the tendency to experience unpleasant emotions easily.

The BFI-10 (18) consists of 10 items, two for each of the five main dimensions of personality. It was designed to economically capture personality according to the five-factor model (Big Five Model) *via* a positive and a negative poled item for each dimension (19, 20). In this work we use one total score per factor (BFI extraversion, BFI agreeableness, BFI conscientiousness, BFI neuroticism, BFI openness).

### NLS training

Trainings were conducted in the afternoon at the Pediatric Simulation Center of the Medical University of Vienna. One training session lasted about 90 min. Every trainings session was suited for eight participants in total to allow enough opportunities for hands on training. First of all, students received a theoretical NLS lecture where an experienced instructor provided a basic understanding of the newborn life support algorithm according to the European Resuscitation Council (ERC) Guidelines 2015 (21). Afterwards, participants trained the algorithm on a Newborn Manikin (Laerdal Medical GmbH, Stavanger, Norway) in different scenarios. Each scenario was performed by two students, and lasted about five minutes. The qualified NLS trainer thereby focused on correct performance of bag mask ventilation as well as chest compressions. Afterwards, every students received a short, structured debriefing from the instructor on how to improve performance in the next scenario. Participants in the control group, however, did not receive any training session prior the assessment scenario.

### Assessment scenario

All selected students (training group vs. control group) had to perform in a pre-defined simulated NLS scenario for assessment purposes. The scenario was unknown to the students. Each student had to take care of a term born neonate (40 + 4 weeks gestational age) with signs of asphyxia immediately after emergency cesarean section due to suspected abruption of the placenta. The infant presented non-viable after birth with a bradycardic heart rate (58/min), so that immediate neonatal resuscitation had to be considered necessary. The main steps of the resuscitation included airway management, chest compressions, calling for help, and the initiation of a venous catheter to administer fluids. The infant's vital parameters were visualized on a simulation monitor.

To avoid any bias due to misunderstandings in communication or teamwork, students participated individually in the scenario and were supported by a study nurse belonging to the study team, who did not interfere in any decision-making processes. However, in case the participant did not decide which step to do next, the study nurse was allowed to give advice so that scenario flow was not interrupted. Each participant received a short, structured debriefing after the assessment.

### Performance

To assess participants' performances in the NLS scenario, we used a scenario-based designed NLS checklist (Supplementary Figure S1). This assessment tool consists of 37 items with 30 items being dichotomous and seven providing more than two categories (e.g., done/insufficiently done/not done), and a maximum total score of 46 points. Content validity of each item was assured by a previous three-round Delphi Process with ten experts in Neonatology. Expert opinion considered values between 46 and 32 as a good/sufficient performance, while scores under 32 were considered as moderate/poor performance. Performance was scored by two co-authors KB and FE independently by assessing the recorded scenario videos.

### Descriptive statistics

For metric parameters, such as raw questionnaires values, we report means ± standard deviations (SD). For nominal (categorical) parameters, such as gender, absolute frequencies as well as frequencies in percent, are specified. For ordinal (categorical) parameters, such as scoring level at a given questionnaire, cumulative frequencies (absolute and relative) are considered.

A two-sample t-test with a two-sided significance level of *α* = 0.05 was used to compare the study groups in their baseline characteristics and raw questionnaire scores, whereas a *χ*^2^-test with a two-sided significance level *α* = 0.05 was applied on the categorical data.

### Machine learning-based statistical analyses

We opted using machine learning-based multivariate statistical analysis over a traditional multivariate analysis as the former overcomes numerous limitations of the latter. Especially as machine learning offers flexible, complex, high dimensional, yet robust, credible, and assessable models (22, 23). These models are not limited or biased by assumptions on variable interactions, by variable scales, or by model oversimplification (22, 23). These are important advancements since such assumptions and simplifications can lead to irrelevant or questionable scientific theories and conclusions (22–24). Three machine learning based analyses were performed [Python v3.9.9, scikit-learn v1.0.2 (25)].

First, a regression analysis to predict internality vs. externality control belief (important to understand how people take responsibility of their action; this is the prediction target) from the following predictors: Trait Anxiety (STAI), Extraversion (BFI), Agreeableness (BFI), Conscientiousness (BFI), Neuroticism (BFI), Openness (BFI), Cognitive reappraisal (ERQ), Expressive suppression (ERQ), and gender.

Second, a regression analysis to predict the participants performance in the NLS scenario (defi points; this is the prediction target) from the following predictors: Trait Anxiety (STAI), BFI extraversion (BFI), BFI agreeableness (BFI), BFI conscientiousness (BFI), BFI neuroticism (BFI), BFI openness (BFI), Cognitive reappraisal (ERQ), Expressive suppression (ERQ), Internality vs. externality control belief (FKK), training group, and gender.

Third a classification analysis to predict good/bad performer in the NLS scenario (scoring *via* experts; this is the prediction target) from the following predictors: Trait Anxiety (STAI), Extraversion (BFI), Agreeableness (BFI), Conscientiousness (BFI), Neuroticism (BFI), Openness (BFI), Cognitive reappraisal (ERQ), Expressive suppression (ERQ), Internality vs. externality control belief, training group, and gender.

#### Machine learning procedure

Extremely randomized Trees (ET) ensembles were chosen to perform the actual prediction tasks (regression as well as classification) because this machine learning method is computationally efficient and highly accurate (26). It can inherently model associations between predictors and prediction targets that go beyond a constant factor (non-linear associations) as well as predictor interactions (26). Furthermore, ET ensembles are robust against multicollinearity and outliers in data (26). Regression performance—the prediction accuracy—was assessed using a nested cross-validation (CV) procedure (27). CV implements repeated train-test splits of the data to assess the generalizability of a model to new unseen data. A shuffle-split scheme (25% of the participants in the testing-set, 75% of the participants in the training-set, 256 repetitions) was applied in the main (outer) CV loop. In each repetition, the training-set was used for data scaling (standardization) and model complexity optimization. Model complexity optimization was carried out in a nested (inner) CV procedure using a sequential Bayesian optimization procedure in combination with a shuffle-split scheme (128 repetition, 64 initial points) to find the best performing complexity parameters (BayesSearchCV, scikit-optimize, v0.9, min_samples_split 2 to total number of samples, min_samples_leaf 1 to total number of samples/2, max_features 1e-1 to 1e0). The complexity parameters that led to the highest prediction accuracy in the training-set of the inner CV (shuffle-split, 25% testing, 75% training, 32 repetitions) were subsequently used along with the constant parameters n_estimators = 512, criterion = friedman_mse and all other parameters left to default, to train regressor models in the main CV loop. The models were subsequently tested on the respective testing-set of the main CV loop. The testing-set was explicitly not used in the inner CV loop.

Regression performance was measured with (1) the prediction coefficient of determination (prediction *R*^2^), as well as with (2) the mean absolute error (28). *R*^2^ values are scaled that 0 equals a performance as good as using the average value of the dependent variable as predictor (the trivial predictor), and that 1 means no error at all. However, *R*^2^ values can have values between minus infinity (a model that performs worse than using the mean value of the dependent variable as the prediction) and 1 (perfect model, no error at all). Notably, the prediction *R*^2^ is expected to be smaller than *R*^2^ values of conventional statistical models because the prediction *R*^2^ measures prediction performance for unknown data and not *post hoc* model fit (28). The mean absolute error reflects the average error that is made at each prediction and is not normalized; hence, it is measured in the scale of the dependent factors.

Classification performance was measured with weighted classification accuracy (28). Since good NLS scenario performer were underrepresented in our sample, their contribution to the accuracy measure was upweighted so that the total contribution of each group (good and bad performers) was equal.

#### Machine learning model analysis

Model analysis, hence, the importance of single predictors for the prediction (regression or classification performance), was carried out with SHAP (SHapely Additive exPlainations) (29). SHAP is a method from interpretable machine learning that is based on Shapley values – a method from cooperative game theory – and measures the contributions of each predictor for a model's total prediction. Pooling those contributions over many predictions allows a comprehensive analysis of the importance of single predictors for the prediction task (29). Today, precise sample size justification for complex, high-dimensional, multivariable models from the machine learning field is still an open matter and no standards have been established. A disputed simple suggestion is that 50 samples are required to start any meaningful machine learning based data analysis (25).

#### Statistical tests

Statistical significance of the prediction performance metrics and of the predictors importance's was assessed using a modified t-test that takes the sample dependence due to the CV into account (30). If necessary, t-test results where Bonferroni corrected for multiple comparisons.

## Results

### Descriptive statistics

We included 602 third-year medical students with a mean age of 22.64 years and a SD of 2.51 with a minimum of 18 years and a maximum of 42 years in the study. Within this collective, 242 (40.2%) were males and 360 females (59.8%). The mean age for men was 23.73 ± 2.74, while for female, it was 22.38 ± 2.32. Among all students, 25 (4.1%) participants had a previous NLS experience (13 were man and 12 female).

The mean STAI-trait total score was 37.29 ± 8.11. Other raw information about the scores obtained from another questionnaire are presented in Table 1.

**Table 1:** Descriptive characteristics: questionnaire raw values.

	*M*	SD
**Personality traits (BFI-10)**		
Extraversion	2.57	0.94
Agreeableness	2.45	0.76
Conscientiousness	2.80	0.80
Neuroticism	1.74	0.96
Openness	2.77	0.97
**Trait Anxiety (STAI)**	37.29	8.11
**Emotional Regulation (ERQ)**		
Cognitive reappraisal	23.31	4.68
Expressive suppression	19.11	3.92
**Locus of Control (FKK)**		
Self concept	34.29	5.63
Internally control beliefs	33.73	4.57
Social externality	24.22	5.15
Fatalistic externality	22.00	5.74
Scala self-efficacy	68.02	8.81
Scala externality	46.22	9.77
Internality Vs Externality	21.80	16.17

BFI, big five-inventory-10; STAI, state-trait anxiety inventory; ERQ, emotional regulation questionnaire; FKK, Fragebogen zu Kompetenz- und Kontrollüberzeugungen (questionnaire for measurement of competence and control).

Subgroup analysis for gender shows differences in almost all psychological characteristics considered except for expressive suppression (ERQ) and internal control beliefs (FKK) (Table 2).

**Table 2:** Descriptive characteristics: psychological traits and characteristics differences between female and male.

	Female		Male		*p*-values	95%CI	
	*M*	SD	*M*	SD		LL	UL
**Personality traits (BFI-10)**							
Extraversion	2.64	0.93	2.46	0.95	0.028	−0.32	−0.01
Agreeableness	2.52	0.76	2.36	0.76	0.015	−0.28	−0.03
Conscientiousness	2.87	0.80	2.70	0.80	0.012	−0.30	−0.03
Neuroticism	1.96	0.92	1.42	0.93	0.000	−0.68	−0.38
Openness	2.84	0.94	2.67	1.01	0.027	−0.33	−0.02
**Trait Anxiety (STAI)**	38.36	8.28	35.68	7.59	0.000	−3.98	−1.36
**Emotional Regulation (ERQ)**							
Cognitive reappraisal	22.94	4.63	23.87	4.71	0.017	0.16	1.68
Expressive suppression	19.21	3.95	18.96	3.86	0.447	−0.88	0.39
**Locus of Control (FKK)**							
Self concept	33.42	5.22	35.59	5.96	0.000	1.26	3.07
Internally control beliefs	33.71	4.35	33.76	4.90	0.887	−0.68	0.80
Social externality	24.81	5.08	23.35	5.14	0.001	−2.29	−0.62
Fatalistic externality	22.64	5.54	21.03	5.91	0.001	−2.54	−0.68
Scala self-efficacy	67.13	8.14	69.35	9.58	0.002	0.79	3.65
Scala externality	47.46	9.40	44.38	10.04	0.000	−4.65	−1.49
Internality Vs Externality	19.67	14.87	24.97	17.50	0.000	2.69	7.91

BFI, big five-inventory-10; STAI, state-trait anxiety inventory; ERQ, emotional regulation questionnaire; FKK, Fragebogen zu Kompetenz- und Kontrollüberzeugungen (questionnaire for measurement of competence and control); LL and UL represents respectively the lower limit and upper limit of the confidence interval.

Particularly, woman had significantly higher values in extraversion (BFI), consciousness (BFI), neuroticism (BFI), and lower values at the subscale internality vs. externality (FKK).Three hundred and twenty-nine participants were reached out by mail from the first recruitment round; for fifty-five of them was possible to organize a simulation scenario. (22 male and 33 female). Previous to the scenario students were randomly assigned to the training group. Twenty-seven (49.1%) received an NLS training before, while 28 did not (control group). None of the participants had previous NLS experience, was a smoker or was regularly using medication. No difference were detected in gender distribution among the groups (15 woman and 13 man in the not training group vs. 18 woman and 9 man in the control group; *p* = 0.412); as well as no difference were detected in age distribution (22.9 ± 2.2 in the not training group vs. 22.8 ± 4.4 in the training group; *p* = 0.906).

**Figure 1:**
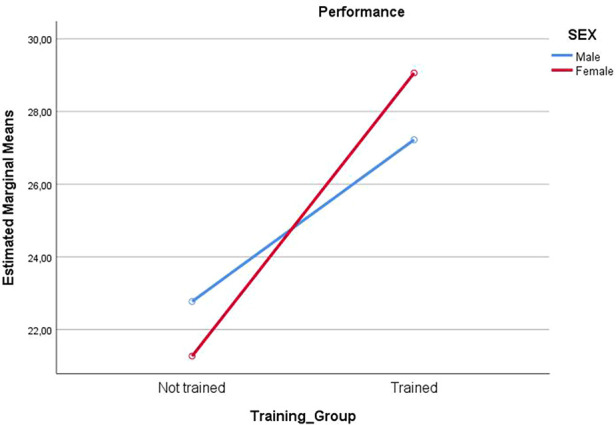
Estimated marginal means considering the effect of training and gender on performance.

Participants’ performance was assessed in the simulated scenario following a checklist based on tasks performed in the scenario as previously described. One participant was excluded because of missing recording material. When looking at the checklist, unadjusted values differences could be found in performance (*p* < 0.001) with the training group showing better performance compared to the no-training group (28.42 ± 4.19 vs. 21.96 ± 4.83). No other statistically significant differences were found for personalities/psychological characteristics between the training group and the no training group. In a univariate model investigating the predictive value of training and gender on performance, training was found to be significant (*p* < 0.001) with an effect of 7.73 points on performance (Figure 1). Neither the effect of gender nor the interaction between gender and training was found to be significant (respectively *p* = 0.914; and *p* = 0.203).

Subsequently, a machine learning based analysis approach was used to understand the effect of different psychological characteristics on internality vs. externality control belief (important to understand how people take responsibility of their action), as well as the impact of training on performance by taking in consideration important personality and psychological elements.

### Machine learning based statistical analyses

#### Regression analysis to predict FFK internality vs. Externality control belief (taking responsibility of own action)

**Figure 2:**
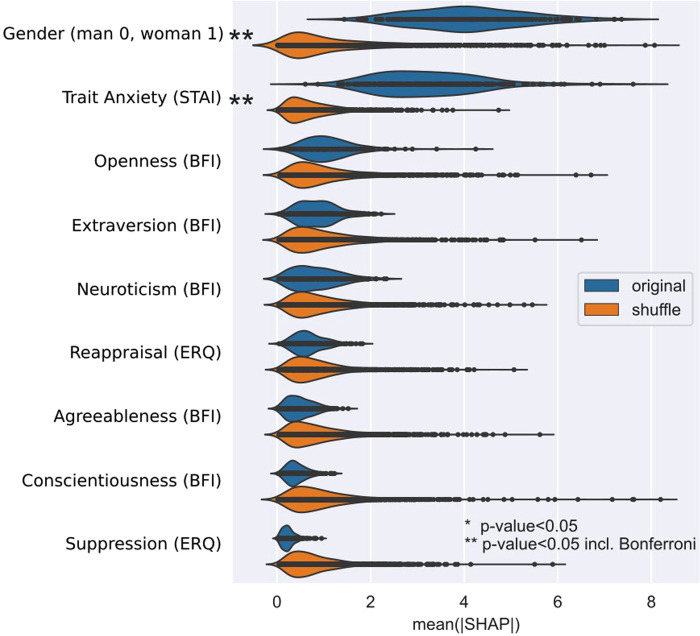
Importance of factors for the prediction of internality vs. externality control belief (FKK_skpc) when using an extra trees (ET) regressor.

The results of the machine learning based multivariate regression analysis with prediction target internality vs. externality control belief (FKK) are summarized in Table 3, first row. About 27% of the variance in control belief can be explained by the regression model that uses Trait Anxiety (STAI), Extraversion (BFI), Agreeableness (BFI), Conscientiousness (BFI), Neuroticism (BFI), Openness (BFI), Cognitive reappraisal (ERQ), Expressive suppression (ERQ), and gender as prediction factors. This is statistically significantly higher than the prediction achieved with shuffled control belief values (*p* = 0.006). On average, the predictions are off by 9.14 points given a control believe range of −80 to 80. The predictor that contributes most for the prediction was gender, followed by Trait Anxiety (Figure 2).

**Table 3:** Summary of the regression and classification results.

Prediction target	*R*^2^ ± std	*p*	MAE ± std	*p*	Acc ± std	*p*
Internality vs. Externality control belief (FKK)	0.27 ± 0.21	0.006	9.14 ± 1.75	0.028	–	–
Performance NLS (defi points)	0.14 ± 0.30	0.054	3.83 ± 0.64	0.054	–	–
Performance NLS (good vs. bad)	–	–	–	–	68.03% ± 16.96	0.159

*R*^2^: prediction coefficient of determination, fraction of explained variance in the prediction target by the machine learning models (sensible for regression only).

MAE: mean absolute error of the models’ predictions (sensible for regression only).

Acc: prediction accuracy, fraction of right classifications (sensible for classification only).

*p*: *p*-values describe how likely these values occur if the null hypothesis is true (shuffle models).

#### Regression analysis to predict participants performance in the NLS scenario

**Figure 3:**
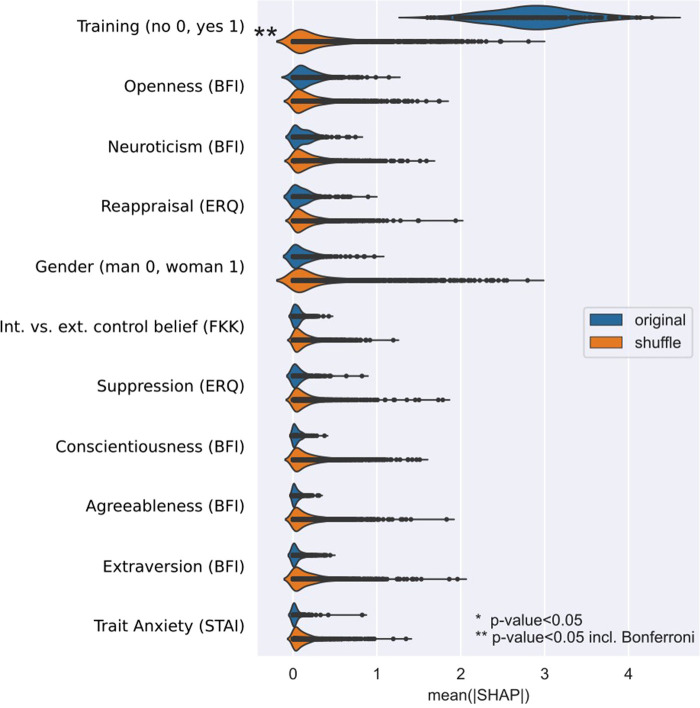
Importance of factors for the prediction of participants performance in the NLS scenario (defi points) when using an extra trees (ET) regressor. Total importance is given by the mean |SHAP| value.

The results of the machine learning based multivariate regression analysis with prediction target participants performance in the NLS scenario (defi points) are summarized in Table 3, second row. About 14% of the variance in participants performance in the NLS scenario can be explained by the regression model that uses Trait Anxiety (STAI), Extraversion (BFI), Agreeableness (BFI), Conscientiousness (BFI), Neuroticism (BFI), Openness (BFI), Cognitive reappraisal (ERQ), Expressive suppression (ERQ), Internality vs. externality control belief, training group, and gender as prediction factors. This result is not statistically significantly higher than the prediction achieved with shuffled defi points values (*p* = 0.054). On average, the predictions are off by about 3.83 points given a defi points range of 0 to 46. The only factor that contributes for the prediction was training (Figure 3).

#### Classification analysis to predict good vs. Bad participant performance in the NLS scenario

**Figure 4:**
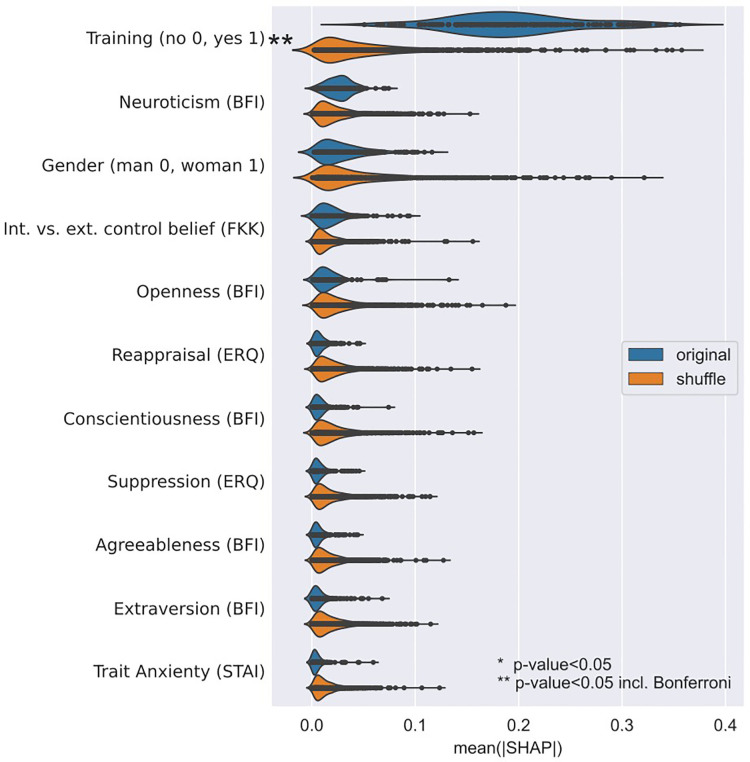
Importance of factors for the prediction of good/bad performer in the NLS scenario when using an extra trees (ET) classifier.

The results of the machine learning based multivariate classification analysis with prediction target good/bad performer in the NLS scenario are summarized in Table 3, third row. Good/bad can be distinguished with about 68% accuracy with the classification model that uses Trait Anxiety (STAI), Extraversion (BFI), Agreeableness (BFI), Conscientiousness (BFI), Neuroticism (BFI), Openness (BFI), Cognitive reappraisal (ERQ), Expressive suppression (ERQ), Internality vs. externality control belief, training group, and gender as prediction factors. This result is not statistically significantly higher than the prediction achieved with shuffled good/bad performance values (*p* = 0.159). The only factor that contributes for the prediction was training (Figure 4).

## Discussion

This study, investigating relationship between personality characteristics and training on resuscitation performance in a simulation setting, revealed that taking responsibility of own action (an element that could be crucial in such demanding scenario) was significantly related to trait anxiety and gender. Even expecting the previous mentioned factor and other psychological characteristics relevant for the resuscitation scenario training was the only variable having an important impact on performance.

### The effect of training

NLS training was found to be the only factor that statistically significantly contributes towards predicting students' performance in the simulated neonatal resuscitation scenario. Due to the study design using a control group and a randomized group assignment, our result not only indicate a correlation, but establish a causal link between training and performance (31).

This result comes not surprisingly since many studies sustain the premise that training improves performance but, in contrast to our study, past research mainly focuses on different training models in different populations of interest, rather than on the relation between acquired experience *via* training, psychological characteristics, and personality traits. Coggins and colleagues, for instance, found an improvement in performance after training for mechanical cardiopulmonary resuscitation (CPR) in medical doctors and nurses following a randomized controlled trial. McCoy and colleagues (32) compared simulation vs. standard training in medical students for cardiopulmonary resuscitation, finding that high-fidelity simulation training was superior to low-fidelity CPR manikin training in term of chest compression depth and compression fraction (32). Other studies also confirmed the effect of training by using different methods such as shared mental models for teams (33), deliberate practice (34), or simply focusing on timing (35).

Evidence from these studies suggests holistic aspects of training, from the cognitive and motor level to the most fundamental theoretical knowledge. The medical students assigned for training in our study received the knowledge in a theoretical part and were trained afterwards on a manikin to achieve basic life support skills that could further help them to properly react in a simulated resuscitation scenario.

The combination of these training sessions was so relevant in our study collective that training was identified among many others as the only factor influencing performance. Nevertheless, we cannot drive firm conclusions, yet, on e.g., how long the effect of training will impact performance, or which type of training is most effective.

In fact, other studies underline the importance of repetition as a key factor to retain skills and knowledge for a longer period of time (35).

### The effect of psychological characteristics and personality traits on performance

Findings from previous studies mostly focused on the link between self-confidence (one's general perceptions of the self in given domains of functioning) and performance in a simulated resuscitation scenario (6, 36–38), as well as the improvement of self-efficacy (person's judgements of his or her capabilities to organize and execute courses of actions) with training (39–41). In our study, we focused on internality vs. externality control belief (FKK), thought understanding how people takes responsibility of own action may play a major role in such a scenario. According to our result, gender (male) and trait anxiety (low values) were the most important predictors associated with this factor.

Anxiety is an extensively studied trait, whose influence on cognition and success has been already largely discussed (42). It is generally seen as a suppressing factor on academic and professional performance as well as a stress trigger. However, anxiety within limits can improve decision making and learning. High level of anxiety correlates usually with the construct of Neuroticism, a personality trait potentially modulating the internal vs. external locus of control, as well as, indirectly, lower performance in medical school students (25).

### Gender, personality traits and leadership

Gender differences in personalities traits have already been described previously, addressing mainly two points of discussion: biological and social. In fact, according to Faingold and colleagues (43), hormonal aspects could reflect innate temperamental differences; while social believes and cultural stereotypes could influence self-expectation and own believes modeling our personality (43). According to previous studies, men are usually described as less anxious and more assertive, while woman have been reported to be more agreeable, nurturing, and altruistic (43, 44). In more, while no differences have been reported for self-esteem and locus of control (43), dissimilarities have been found in the BIG-five questionnaire. Similar to us, Weisberg and colleagues (44) have found gender differences for: neuroticism, agreeableness, and extraversion. Neuroticism, a factor that describe the tendency to experience negative emotion, was found to be higher in adult women (having age as mediator factor) as also supported by our data. Also, agreeableness, as in our data, was found to be higher in woman, reflecting a higher altruism and empathy. Lastly, extroversion was found to be significant between the groups, were women scored higher than men in sociability, but not in assertiveness which reflects traits of agency and dominance (44). Since we used a reduced item version of the Big-five, we cannot replicate on this point.

Personality traits may reflect a given leadership character having an impact on team performance. However, different context may profit from different type of leadership, making difficult to generalize results (45).

Tremèr and colleagues (46), In a study that had the goal to investigate how different personality traits, self-esteem levels and leadership assignment of medical students impact performance, physiological stress responses and perceived stress during a resuscitation simulation, were able to demonstrate that in groups without a designated leader, conscientious and confident students were more likely to have the longest period of uninterrupted resuscitation (hands-on time), whereas in groups with a leader, an agreeable leader was the key to a sustained hands-on time. An agreeable leader was therefore associated with a more sustained hands-on time whereas neuroticism was linked to insufficient leadership (46).

### Limitation

The statistical power of our regression analyses is relatively low given the total number of 55 participants. We expect that with higher statistical power the *p*-value obtained for the model fit in the NLS scenario performance prediction will be below 5%. Nonetheless, the predictors' importance was significant. Our examined psychological characteristics and personality traits did not describe the full variance in the NLS scenario performance, hence, there must exist other prediction factors that we did not consider here and that are further potentially important. Unfortunately, there is no gold standard available on how to establish performance in a neonatal resuscitation scenario. We used item related checklist based on aDelphi process. Finally, during the scenario, a study nurse played his role always in the most standard way possible, but we cannot exclude subjective bias in the scenario.

## Conclusion

According to our results, gender and trait anxiety were the most important predictors associated with taking responsibility of an action. In addition, training had a significantly stronger effect on performance in a neonatal resuscitation scenario as compared to psychological characteristics or personality traits. Therefore, it can be emphasized that with regular training everyone in the field of neonatology can improve resuscitation skills, regardless his or her personal characteristics.

## Data Availability

The datasets presented in this article are not readily available because all data generated or analyzed during this study are included in this article [and/or] its **Supplementary Material** files. Further inquiries can be directed to the corresponding author. Requests to access the datasets should be directed to vito.giordano@meduniwien.ac.at.

## References

[1] Vilar E Rebelo F Noriega P Duarte E Mayhorn CB Effects of competing environmental variables and signage on route-choices in simulated everyday and emergency wayfinding situations. Ergonomics. (2014) 57(4):511–24. 10.1080/00140139.2014.895054 24635043

[2] Jeong J Hong KJ Shin SD Ro YS Song KJ Lee EJ Lee YJ Ahn KO Relationship between drowning location and outcome after drowning-associated out-of-hospital cardiac arrest: nationwide study. Am J Emerg Med. (2016) 34(9):1799–803. 10.1016/j.ajem.2016.06.008 27342967

[3] Barcala-Furelos R Szpilman D Palacios-Aguilar J Costas-Veiga J Abelairas-Gomez C Bores-Cerezal A Lopez-Garcia S Rodriguez-Nunez A Assessing the efficacy of rescue equipment in lifeguard resuscitation efforts for drowning. Am J Emerg Med. (2016) 34(3):480–5. 10.1016/j.ajem.2015.12.006 26782793

[4] Hunziker S Johansson AC Tschan F Semmer NK Rock L Howell MD Marsch S Teamwork and leadership in cardiopulmonary resuscitation. J Am Coll Cardiol. (2011) 57(24):2381–8. 10.1016/j.jacc.2011.03.017 21658557

[5] Barcala-Furelos R Abelairas-Gomez C Romo-Perez V Palacios-Aguilar J Effect of physical fatigue on the quality CPR: a water rescue study of lifeguards: physical fatigue and quality CPR in a water rescue. Am J Emerg Med. (2013) 31(3):473–7. 10.1016/j.ajem.2012.09.012 23085007

[6] Peacock PJ Woodman A McCay W Bates SE Resuscitation of the newborn: simulating for confidence. Cureus. (2016) 8(9):e790. 10.7759/cureus.790 27774358 PMC5072661

[7] Guise JM Hansen M O'Brien K Dickinson C Meckler G Engle P Lambert W Jui J Emergency medical services responders’ perceptions of the effect of stress and anxiety on patient safety in the out-of-hospital emergency care of children: a qualitative study. BMJ Open. (2017) 7(2):e014057. 10.1136/bmjopen-2016-014057 28246139 PMC5337745

[8] Schmolzer GM M OR Fray C van Os S Cheung PY Chest compression during sustained inflation versus 3:1 chest compression:ventilation ratio during neonatal cardiopulmonary resuscitation: a randomised feasibility trial. Arch Dis Child Fetal Neonatal Ed. (2017) pii:fetalneonatal-2017-313037. 10.1136/archdischild-2017-313037 28988159

[9] Madar J Roehr CC Ainsworth S Ersdal H Morley C Rüdiger M Skåre C Szczapa T Te Pas A Trevisanuto D European Resuscitation council guidelines 2021: newborn resuscitation and support of transition of infants at birth. Resuscitation. (2021) 161:291–326. 10.1016/j.resuscitation.2021.02.014 33773829

[10] McKay KA Buen JE Bohan KJ Maye JP Determining the relationship of acute stress, anxiety, and salivary alpha-amylase level with performance of student nurse anesthetists during human-based anesthesia simulator training. AANA J. (2010) 78(4):301–9. PMID: 2087963 20879631

[11] LeBlanc VR. The effects of acute stress on performance: implications for health professions education. Acad Med. (2009) 84(10 Suppl):S25–33. 10.1097/ACM.0b013e3181b37b8f 19907380

[12] Cutuli D. Cognitive reappraisal and expressive suppression strategies role in the emotion regulation: an overview on their modulatory effects and neural correlates. Front Syst Neurosci. (2014) 8:175. 10.3389/fnsys.2014.00175 25285072 PMC4168764

[13] Krampen G. FKK – Fragebogen Zu Kompetenz- Und Kontrollüberzeugungen. Göttingen: Hogrefe (1991).

[14] McCrae RR Costa PT. Validation of the five-factor model of personality across instruments and observers. J Pers Soc Psychol. (1987) 52(1):81–90. 10.1037/0022-3514.52.1.81 3820081

[15] Spielberger CD. State-trait anxiety inventory, in the corsini encyclopedia of psychology. John Wiley & Sons, Inc. (2010). 10.1002/9780470479216.corpsy0943

[16] Abler B Kessler H. Emotion regulation questionnaire – eine deutschsprachige fassung des ERQ von gross und john. Diagnostica. (2009) 55(3):144–52. 10.1026/0012-1924.55.3.144

[17] Gross JJ John OP. Individual differences in two emotion regulation processes: implications for affect, relationships, and well-being. J Pers Soc Psychol. (2003) 85(2):348–62. 10.1037/0022-3514.85.2.348 12916575

[18] Rammstedt B. The 10-item big five inventory. Eur J Psychol Assess. (2007) 23(3):193–201. 10.1027/1015-5759.23.3.193

[19] Raad BD. Five big, big five issues. Eur Psychol. (1998) 3(2):113–24. 10.1027/1016-9040.3.2.113

[20] Wiggins JS Trapnell PD. Chapter 28 - personality structure: the return of the big five. In: Hogan R Johnson J Briggs S, editors. Handbook of personality psychology. San Diego: Academic Press (1997). p. 737–65.

[21] European Resuscitation Council. European Resuscitation Council Guidelines for Resuscitation (2015): Section 6. *Paediatric Life Support*. Available at: https://ercguidelines.elsevierresource.com/european-resuscitation-council-guidelines-resuscitation-2015-section-6-paediatric-life-support (cited February 23, 2022). 10.1016/j.resuscitation.2015.07.028 26477414

[22] Breiman L. Statistical modeling: the two cultures (with comments and a rejoinder by the author). Stat Sci. (2001) 16(3):199–231. 10.1214/ss/1009213726

[23] Westreich D Greenland S. The Table 2 fallacy: presenting and interpreting confounder and modifier coefficients. Am J Epidemiol. (2013) 177(4):292–8. 10.1093/aje/kws412 23371353 PMC3626058

[24] Jolly E Chang LJ. The flatland fallacy: moving beyond low–dimensional thinking. Top Cogn Sci. (2019) 11(2):433–54. 10.1111/tops.12404 30576066 PMC6519046

[25] Pedregosa F Varoquaux G Gramfort A Michel V Thirion B Grisel O Blondel M Müller A Nothman J Louppe G Scikit-learn: Machine Learning in Python. (2012). arXiv:1201.0490).

[26] Geurts P Ernst D Wehenkel L. Extremely randomized trees. Mach Learn. (2006) 63(1):3–42. 10.1007/s10994-006-6226-1

[27] Cawley GC Talbot NLC. On over-fitting in model selection and subsequent selection bias in performance evaluation. J. Mach. Learn. Res. (2010) 11:2079–107. http://jmlr.csail.mit.edu/papers/v11/cawley10a.html

[28] Hastie T Tibshirani R Friedman J. The elements of statistical learning. Springer series in statistics. New York, NY: Springer (2009). Vol. 2. XXII. 745 p.

[29] Lundberg S Lee S-I. A Unified Approach to Interpreting Model Predictions. (2017). arXiv:1705.07874).

[30] Nadeau C Bengio Y. Inference for the generalization error. Mach Learn. (2003) 52(3):239–81. 10.1023/A:1024068626366

[31] Marinescu IE Lawlor PN Kording KP. Quasi-experimental causality in neuroscience and behavioural research. Nat Hum Behav. (2018) 2(12):891–8. 10.1038/s41562-018-0466-5 30988445

[32] McCoy CE Rahman A Rendon JC Anderson CL Langdorf MI Lotfipour S Chakravarthy B Randomized controlled trial of simulation vs. Standard training for teaching medical students high-quality cardiopulmonary resuscitation. West J Emerg Med. (2019) 20(1):15–22. 10.5811/westjem.2018.11.39040 30643596 PMC6324716

[33] Beck S Doehn C Funk H Kosan J Issleib M Daubmann A Zöllner C Kubitz JC Basic life support training using shared mental models improves team performance of first responders on Normal wards: a randomised controlled simulation trial. Resuscitation. (2019) 144:33–9. 10.1016/j.resuscitation.2019.08.040 31505232

[34] Hunt EA Duval-Arnould JM Nelson-McMillan KL Bradshaw JH Diener-West M Perretta JS Shilkofski NA Pediatric resident resuscitation skills improve after “rapid cycle deliberate practice” training. Resuscitation. (2014) 85(7):945–51. 10.1016/j.resuscitation.2014.02.025 24607871

[35] Anderson R Sebaldt A Lin Y Cheng A Optimal training frequency for acquisition and retention of high-quality CPR skills: a randomized trial. Resuscitation. (2019) 135:153–61. 10.1016/j.resuscitation.2018.10.033 30391370

[36] Blewer AL Leary M Esposito EC Gonzalez M Riegel B Bobrow BJ Abella BS Continuous chest compression cardiopulmonary resuscitation training promotes rescuer self-confidence and increased secondary training: a hospital-based randomized controlled trial*. Crit Care Med. (2012) 40(3):787–92. 10.1097/CCM.0b013e318236f2ca 22080629 PMC3746171

[37] Labrague LJ McEnroe-Petitte DM Bowling AM Nwafor CE Tsaras K High-fidelity simulation and nursing students’ anxiety and self-confidence: a systematic review. Nurs Forum. (2019) 54(3):358–68. 10.1111/nuf.12337 30852844

[38] Sergeev I Lipsky AM Ganor O Lending G Abebe-Campino G Morose A Katzenell U Ash N Glassberg E Training modalities and self-confidence building in performance of life-saving procedures. Mil Med. (2012) 177(8):901–6. 10.7205/MILMED-D-12-00018 22934367

[39] Ein-Gar D Steinhart Y. Self-control and task timing shift self-efficacy and influence willingness to engage in effortful tasks. Front Psychol. (2017) 8:1788. 10.3389/fpsyg.2017.01788 29075225 PMC5641896

[40] Maibach EW Schieber RA Carroll M. Self-efficacy in pediatric resuscitation: implications for education and performance. Pediatrics. (1996) 97(1):94–9. 10.1007/s10459-011-9274-7 8545233

[41] Plant JL van Schaik SM Sliwka DC Boscardin CK O'Sullivan PS Validation of a self-efficacy instrument and its relationship to performance of crisis resource management skills. Adv Health Sci Educ. (2011) 16(5):579–90. 10.1007/s10459-011-9274-7 PMC3226693 21264508

[42] Hardy L. Stress, anxiety and performance. J Sci Med Sport. (1999) 2(3):227–33. 10.1016/S1440-2440(99)80175-3 10668760

[43] Feingold A. Gender differences in personality: a meta-analysis. Psychol Bull. (1994) 116(3):429. 10.1037/0033-2909.116.3.429 7809307

[44] Weisberg Y DeYoung C Hirsh J. Gender differences in personality across the ten aspects of the big five. Front Psychol. (2011) 2. 10.3389/fpsyg.2011.00178 21866227 PMC3149680

[45] O'Donovan R Rogers L Khurshid Z De Brún A Nicholson E O'Shea M Ward M McAuliffe E A systematic review exploring the impact of focal leader behaviours on health care team performance. J Nurs Manag. (2021) 29(6):1420–43. 10.1111/jonm.13403 34196046

[46] Tramèr L Becker C Schumacher C Beck K Tschan F Semmer NK Hochstrasser S Marsch S Hunziker S Association of self-esteem, personality, stress and gender with performance of a resuscitation team: a simulation-based study. PLoS One. (2020) 15(5):e0233155. 10.1371/journal.pone.0233155 32407382 PMC7224528

