# Supplementary material for: Relationship between psychological characteristics, personality traits, and training on performance in a neonatal resuscitation scenario: A machine learning based analysis
Source: Front Pediatr. 2022 Nov 18;10:1000544. doi: 10.3389/fped.2022.1000544 (PMC9715966; doi:10.3389/fped.2022.1000544)
Supplement: Supplementary file 1 [file Datasheet1.pdf]

NeoCheck

Preparation

Question	Points 0	1	2	3
1 Did the participant put on gloves?	○ not done	○ done		
2 Did the participant turn on the overhead warmer?	○ not done	○ done		
3 Did the participant place at least two warm towels at the resuscitation unit?	○ not done	○ done		
4 Did the participant place the ventilation device (e.g. bag mask valve or T-piece) at the resuscitation unit + checked functionality of the device?	○ not done	○ placed the ventilation device at the resuscitation unit	○ placed the ventilation device at the resuscitation unit & checked functionality	
5 Did the participant check whether the mask size is appropriate for the estimated weight of the newborn?	○ not done	○ done		
6 Did the participant test and prepare the suction unit?	○ not done	○ prepared or tested suction unit	○ prepared & tested suction unit	
7 Did the participant place the ECG leads to assess basic heart rate at the resuscitation unit?	○ not done	○ done		
8 Did the participant connect the oxygen tubing to the ventilation device (e.g. bag-mask valve or T-piece)?	○ not done	○ done		
9 Did the participant place the oxygen saturation probe at the resuscitation unit?	○ not done	○ done		
10 Did the participant place the stethoscope at the resuscitation unit?	○ not done	○ done		
11 Did the participant prepare for any additional specific interenventions such as intravenous access, fluid, medication?	○ not done	○ done		

NeoCheck

Resuscitation (1/2)	Question	Points 0	1	2	3
	12 Did the participant start an available timer at the newborn's arrival at the heat unit?	○ not done	○ done (within 1 min. of arrival)		
	13 Did the participant place the newborn with its head towards him- or herself before ventilation started?	○ not done	○ as ventilation started	○ initially	
	14 Did the participant start with stimulation and drying the newborn?	○ not done	○ drying (because that is also a way of stimulation)	○ drying + extra stimulation (study nurse is advised to rub the feet/body	
	15 Did the participant assess the newborn's tone?	○ not done	○ done		
	16 Did the participant assess whether the baby was breathing?	○ not done		○ done	
	17 Did the participant place the baby´s head in a neutral position?	○ not done	○ done		
	18 Did the participant assess or ask if the airway was obstructed?	○ not done	○ done		
	19 Did the participant start with 5 initial positive pressure ventilations?	○ not done	○ done		
	20 Did the participant assess whether there was chest wall movement with the first 5 initial ventilations?	○ not done	○ done		
	21 Did the participant try to optimize ventilation?	○ not done	○ done alternatives (2-person airway control, repositioning)	○ done (guedel or change of mask size)	○ done alternative + guedel/mask
	22 Did the participant perform another 5 ventilations after having optimized the ventilation before?	○ not done	○ done		
	23 Did the participant assess chest movement again during the second course of 5 ventilations?	○ not done	○ done		

NeoCheck	Question	Points			
		0	1	2	3
Resuscitation (2/2)	24 Did the participant assess the newborns heart rate (e.g. via ECG, auscultation, etc.?	○ not done	○ done		
	25 Did the participant perform another 5 ventilations after having assessed the low heart rate	○ not done	○ done	○ initially	
	26 Did the participant assess chest wall movement during this time of ventilation?	○ not done	○ done		
	27 Did the participant assess heart rate after 2x5 times effective ventilation?	○ not done	○ done		
	28 Did the participant ask for additional help?	○ not done	○ after CPR begin	○ before CPR begin	
	29 Did the participant initiate to start CPR?	○ not done	○ done		
	30 Did the participant use the correct CPR algorithm?	○ not done (or other than 3:1)	○ done (3 compressions: 1 breath)		
	31 Did the participant assess the heartrate after 30 seconds of CPR?	○ not done	○ done +/- 10 seconds	○ done (in time)	
	32 Did the participant stop chest compressions for heart rate assessment?	○ not done	○ done		
	33 Did the participant make the right decision whether to stop or to continue chest compressions? (with HR > 60/min. stop compression)	○ not done	○ done		
	34 Did the participant assess the newborn's breathing after chest compression was ceased?	○ not done	○ done		
	35 Did the participant continue with assisted ventilation?	○ not done	○ done		
	36 Did the participant substitute the wet towels by dry ones at any time during resuscitation?	○ not done	○ done		
	37 Did the participant obtain intravenous or intraosseus access?	○ not done	○ done		
	OVERALL SCORE:				
